# An Alignment Method for the Integration of Underwater 3D Data Captured by a Stereovision System and an Acoustic Camera

**DOI:** 10.3390/s16040536

**Published:** 2016-04-14

**Authors:** Antonio Lagudi, Gianfranco Bianco, Maurizio Muzzupappa, Fabio Bruno

**Affiliations:** DIMEG, University of Calabria, Via P. Bucci 46/C–Rende, Cosenza 87036, Italy; antonio.lagudi@unical.it (A.L.); gianfranco.bianco@unical.it (G.B.); muzzupappa@unical.it (M.M.)

**Keywords:** underwater 3D imaging, opto-acoustic vision, optical and acoustic integration, ROV navigation

## Abstract

The integration of underwater 3D data captured by acoustic and optical systems is a promising technique in various applications such as mapping or vehicle navigation. It allows for compensating the drawbacks of the low resolution of acoustic sensors and the limitations of optical sensors in bad visibility conditions. Aligning these data is a challenging problem, as it is hard to make a point-to-point correspondence. This paper presents a multi-sensor registration for the automatic integration of 3D data acquired from a stereovision system and a 3D acoustic camera in close-range acquisition. An appropriate rig has been used in the laboratory tests to determine the relative position between the two sensor frames. The experimental results show that our alignment approach, based on the acquisition of a rig in several poses, can be adopted to estimate the rigid transformation between the two heterogeneous sensors. A first estimation of the unknown geometric transformation is obtained by a registration of the two 3D point clouds, but it ends up to be strongly affected by noise and data dispersion. A robust and optimal estimation is obtained by a statistical processing of the transformations computed for each pose. The effectiveness of the method has been demonstrated in this first experimentation of the proposed 3D opto-acoustic camera.

## 1. Introduction

Acoustic and optical 3D systems are widely used to collect 3D information in underwater applications, such as 3D reconstruction of submerged archaeological sites, seabed mapping, or Remotely Operated underwater Vehicle (ROV) navigation [1,2,3]. Acoustic systems typically give good results in long-range acquisitions and do not suffer from turbidity, but the resulting 3D data are affected by low resolution and accuracy. Optical systems, in contrast, are more suited for close-range acquisitions and allow for gathering high-resolution, accurate 3D data and textures, but the results are strongly influenced by the visibility conditions. Therefore, the integration of 3D data captured by these two types of systems is a promising technique in underwater applications, as it allows for compensating their respective limitations.

Despite the difficulty of combining two modalities that operate at different resolutions, the integration of optical and acoustic systems in an underwater environment has received increasing attention over the past few years, mainly for seabed mapping and egomotion estimation of underwater vehicles [4,5,6,7,8,9]. Further examples of opto-acoustic integration concern with local area imaging rather than the creation of large area maps. In [10,11,12], the integration of video and 3D data, acquired through a single optical camera and a 3D acoustic camera (Echoscope 1600) [13], is obtained by geometrically registering such data with respect to a well-known model of the observed scene, while in [14,15,16,17,18] the authors propose a new paradigm of opto-acoustic stereo reconstruction that aims to apply the epipolar geometry to a stereo system composed by an optical camera and a 2D sonar (DIDSON).

Up to now, the works presented in the literature about the integration between several types of sonar (single beam sounder, multibeam, 3D acoustic camera) and optical cameras, adopt a sensor fusion approach, which is mapping-oriented, according to the classification proposed in [19]. This means that the data acquired from the two sensors are described through geometric relationships (position and orientation), and the data integration is performed by means of geometrical correspondences and registration. Data alignment, that is, their transformation from each sensor’s local frame into a common reference frame, is a crucial problem of these methods that is usually solved by performing the exterior orientation of the integrated system, *i.e.*, by searching for the rigid transformation between the coordinate systems related to each sensor.

Few works have explicitly treated the alignment problem of opto-acoustic underwater systems. They showed that the methods are highly dependent on the layout and sensors that compose the system, in particular for the type of data structure they provide. In [16], the exterior orientation of the optical camera and the 2D sonar is performed by using a planar grid characterized by considerable optical and acoustic features that are manually associated. Therefore, the relative positions of the cameras are estimated through an optimization algorithm that minimizes the distances between 3D reconstructions of optical and acoustic matching projections.

In [20], the authors propose a method for aligning a single camera with a multibeam sonar on an Autonomous Underwater Vehicle (AUV) using a target placed in a test tank. Assuming a simplified model for the multibeam sonar, the exterior orientation of the proposed opto-acoustic system was compared to the alignment of a laser-camera system, where the method presented in [21] was adopted for its solution. 

Finally, a different methodology is presented in [12]. After some pre-processing steps, the acoustic data are registered with respect to a CAD model of a target (an oil rig in this case) using the Iterative Closest Point (ICP) algorithm [22], while the optical alignment is performed by means of the algorithm proposed in [23]. Once the poses of both sensors are calculated with respect to the observed object, the relative pose between the optical camera and the acoustic camera can be estimated without the need to use positioning and motion system equipment.

The Echoscope 3D acoustic camera is an interesting sonar which, unlike other sonars, ensonifies a whole viewing volume with a single ping and outputs 3D data in real-time as 3D point clouds. Therefore, it is suitable to be coupled with optical devices as it provides whole field 3D data from a single acquisition, unlike other devices such as multibeam sonars that acquire multiple slices and stitches them together according to navigation data. In previous works, 3D acoustic cameras have been used for on-line 3D reconstruction of underwater environments from multiple range views [24], or coupled with a single camera to improve the understanding of the underwater scenes and assist the ROV pilots during the navigation [10,11,12].

In the present work, for the first time, the same 3D acoustic camera used in [10,11,12] has been coupled with a stereovision system to gather synchronous 3D data and perform 3D opto-acoustic imaging of the acquired underwater scene. The system was conceived to improve the understanding of a human operator guiding an underwater ROV during the navigation in variable turbid water conditions and in operations that require the use of one or two manipulators. The stereo optical camera allows for obtaining a better perception of the scene depth if compared to the use of a single camera as in the actual ROV configuration, while the acoustic camera makes its best contribution in poor visibility conditions. Compared to the similar setup previously described in literature [10,11,12], the novelty of the proposed approach lies in the adoption of a stereo optical camera that give us the possibility to have a better resolution of the 3D image when the visibility conditions are good enough for optical sensors; moreover, it allows us to overcome the problem of processing heterogeneous data gathered from different sensors by simplifying the correspondence determination in a registration problem of the two 3D point clouds.

The aim of this work is to solve the problem of the automatic alignment of the optical and acoustic 3D images through the definition of a multi-sensor registration approach [25]. The core idea of the method is:

1. obtaining a raw estimation of the unknown geometric transformation through a registration of the optical and acoustic 3D data for each pose of a custom orientation fixture;
2. obtaining a robust and optimal estimation through a statistical processing of the transformations computed for each pose.

Experimental tests have been conducted in laboratory to validate the feasibility and the effectiveness of the proposed method and quantify the accuracy of the integration. These also gave us the opportunity to perform a first experimentation of the proposed 3D opto-acoustic camera, allowing for a better understanding of limitations and drawbacks of the system, and of the problems related to the alignment itself. The experimental results show that our alignment approach, based on several pose acquisitions of an appropriate rig, can be adopted to simultaneously calibrate the stereo optical system and estimate the rigid transformation between the optical and acoustic sensors. The effectiveness of the method has been demonstrated in this first experimentation of the proposed 3D opto-acoustic camera.

## 2. Relative Orientation of the Opto-Acoustic 3D Camera

To effectively integrate and fuse spatial data from different 3D sensors, the relative position and orientation of their spatial coordinate systems have to be known. The estimate of such spatial relationships can be broken down into two tasks: interior orientation, where internal sensor parameters are determined, and relative orientation, where the position and the orientation of a sensor relative to a given coordinate system are determined.

Assuming that a point $p_{o}={\left[x_{o},y_{o},z_{o}\right]}^{T}$ of the stereo-optical reference frame corresponds to a point $p_{a}={\left[x_{a},y_{a},z_{a}\right]}^{T}$ of the acoustic reference frame, the main goal of our multi-sensor alignment is to determine the rigid transformation Tao that relates the two coordinate systems. It may be expressed, in homogeneous coordinates, as:

(1) po˜=[Rt01]ao pa˜=Tao pa˜

where ***R*** is the orthonormal 3 × 3 matrix that represents the orientation of the acoustic camera to the stereo-optical one, while ***t*** is a three-dimensional vector corresponding to their relative positions.

Our multi-sensor alignment method operates in the following way:

1. it executes a synchronous optical and acoustic acquisition of a fixture in several poses;
2. it calculates both the interior and exterior orientation of the stereo optical system;
3. for each pose of the fixture, it calculates the rigid transformation that relates the sensor reference frames among each other through a registration algorithm;
4. it processes the transformation matrices through statistical methods;
5. it calculates the best estimation for the unknown transformation matrix.

### 2.1. Fixture Design

In the case of a stereo-optical system, the alignment problem can be solved by the optimization of a series of equations in which a collection of correspondent 3D points in both cameras is known. Typically, these data are generated by imaging a fixture that represents a set of 3D points belonging some feature of the target. For example, the centers of a dot pattern and the corners of a checkerboard pattern were used in [26,27], respectively. Such a method is difficult to adopt in the opto-acoustic alignment, because a point-to-point correspondence between 3D points in both representations is not a simple task, *i.e.*, the low resolution and the strong noise component of acoustic data do not allow for precisely localizing a point position as determined for the optical camera, as demonstrated in [2,20]. Therefore, it is necessary to find other features fit for establishing the correspondence between optical and acoustic frames. 

Photogrammetric methods that employ a stereo setup acquire the scene by means of two optical cameras with known internal geometric characteristics (principal point, principal distance, and distortion function) and known relative orientation to each other. These are obtained through calibration, according to the selected camera model. Although in underwater environment the well-known pinhole camera limits the reliability and accuracy of the obtained results, as the effects of refraction must be corrected (or modeled) to obtain an accurate calibration [28], we have chosen to use the method proposed in [27] to calibrate the stereovision system. The main advantages of this approach are the simplicity of the calibration fixture and the rapid measurement and processing of the captured images, made possible by the automatic recognition of the checkerboard pattern. However, as reported in [28], this calibration procedure is suitable for applications with modest accuracy requirements, like in our case.

Taking into account that the reflective properties of the optical and acoustic signals vary according to the materials to be used, we have designed a fixture that has to satisfy the following requirements: (a) it allows for detecting geometric features in both systems; (b) it is able to discriminate or highlight areas on the rig; (c) it can be used for both opto-acoustic alignment and optical stereo calibration, simultaneously. Moreover, our underwater fixture has to satisfy several application-specific requirements, including superior visibility of the calibration markers representing object space points, water resistance of the frame and provisions for convenient deployment and retrieval.

**Figure 1:**
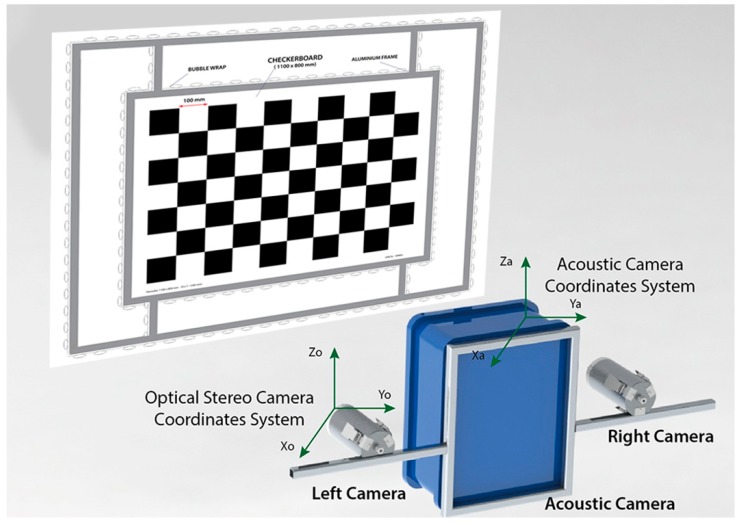
Geometry of the alignment method.

The fixture is composed by a checkerboard panel in the center, built from a thin sheet of aluminum Dibond^®^ (an acoustically transparent material) and fixed on an aluminum frame to calibrate the optical cameras, then another aluminum frame is placed around the inner frame to concentrate the acoustic detection along the bars. To highlight the rig areas to be detected form the acoustic system, we have thought to exploit the high reflectivity of the air in water, so the aluminum bars were covered by bubble wrap. The designed rig allows for referring both 3D data on a known-size frame and to determine simple features as centroid, normal to the plane, orientation, perimeter, edges (Figure 1).

As will be described in Section 3.2, the size of the rig (2 × 2.5 m) was determined through the analysis of the Fields-Of-Views (FOVs) of the sensors in the expected operative range. Although a 3D fixture could be used to obtain a more accurate results, we have chosen to use a 2D rig for our approach, and to move it in a controlled volume. This choice is motivated by the difficulty to handle and move a three-dimensional structure of such size and with the requirements described above, especially in real conditions. 

### 2.2. Optical and Acoustic Data Registration

Since methods that rely on explicit opto-acoustic correspondences have to be avoided [2,20], in our approach the acoustic 3D point clouds representing the orientation rig are matched to the optical counterpart by using the Iterative Closest Point (ICP) algorithm, an iterative least-square technique used for the registration of rigid 3D shapes. This approach eliminates the need to perform any feature extraction or to specify any explicit feature correspondence.

The basic idea behind the ICP algorithm is that, under certain conditions, the point correspondences provided by sets of closest points are reasonable approximations for the true point correspondences. If the process of finding the closest-point sets and then solving the least-square function is iterated, the solution will converge to a local minimum, but there is no guarantee that this will correspond to the actual global minimum.

In our solution, the global convergence is achieved through a data pre-processing to clean up the 3D point clouds from noise or potential outliers, and a coarse registration stage that gives a fairly good initial alignment of the two 3D point clouds.

Therefore, considering the pair $P_{o}P_{a}$ of optical and acoustic 3D point clouds, respectively, the associated Tao is determined as a composition of transformations obtained through a coarse and fine registration technique.

Taking as a reference system the local reference frame of the stereo optical camera, the coarse registration stage was carried out through two operations:

1. calculation of Ta1 by the orientation of the acoustic camera local reference frame, in such a way that the Z axis represents the depth of the scene, in line with the optical system; this step is necessary because the data acquired by the Echoscope are represented in a local reference system in which the depth of the scene is expressed along the *Y* axis;
2. rough alignment of the pair of 3D point clouds $P_{o}P_{a}$ that, through an estimate of the centroid of the two 3D point clouds, determines the translation vector ***t*** that relates them (assuming that the rotation matrix ***R*** is unitary). As a result of this operation, we obtain the transformation matrix T12.

**Figure 2:**
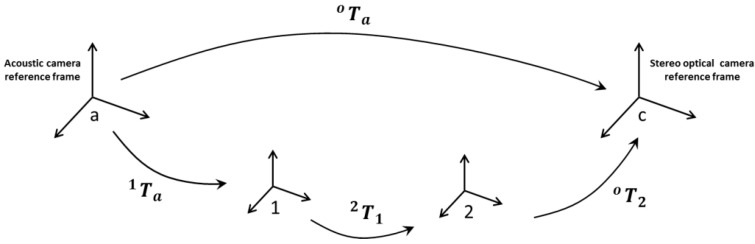
Sequence of transformations for computing the Tao matrix.

Concerning the step of fine registration, a Matlab^®^ implementation of the ICP algorithm has been applied to the pair $P_{o}P_{a}$ aligned in the previous step, to obtain the transformation matrix T2o. Downstream of the previous operations, the unknown rigid transformation matrix Tao is obtained as (Figure 2):

(2) Tao= T2o×T12×Ta1

### 2.3. Statistical Estimation of the Geometric Transformation

From the data registration step described in Section 2.2, we have obtained a first estimation of the unknown geometric transformation that is strongly affected by noise and data dispersion. To perform a robust estimation as the working distance changes, we have decided to acquire several data of the transformation for outputting the optimal estimation through a statistical approach.

Three different methods of statistical processing have been implemented and subsequently compared, to estimate the rigid transformation matrix Ta*o from the *n* matrices Ta,no obtained downstream of the operation of coarse and fine registration applied to *n* pairs $P_{0,n}P_{a,n}$ of 3D point clouds.

As a first hypothesis, we tried to obtain the elements of the final transformation matrix Ta*o through an average calculation on the Ta,no matrices included in the dataset. However, this operation, if applied directly on the homogeneous matrices Ta,no, compromises the orthonormality of the result, so we decided to apply this solution representing the Ta,no in the corresponding $R_{n}$ rotation matrices and $t_{n}$ translation vectors.

Subsequently, as the mean rotation matrix $\bar{R}$ and the mean translation vector $\bar{t}$ were obtained, we put the final transformation matrix Ta*o=[R¯t¯ 01]a*o. The mean translation vector $\bar{t}$ was calculated as a simple arithmetic mean on the elements of the vectors $t_{n}$. Concerning the calculation of the mean rotation matrix $\bar{R}$, there could be multiple solutions. In fact, as reported in [29], there are several formulations in the literature to obtain the mean rotation matrix, either based on Euclidean or Riemannian distance metrics. The same paper also shows how, where data does not present a high variability (as it can be assumed for the present case, since the Ta,no are estimates of the same transformation matrix), the arithmetic mean applied to the rotation vectors $\varphi_{n}$, obtained in turn from the corresponding rotation matrices $R_{n}$, represents an approximate solution to both the Riemannian ${\bar{R}}_{Riem}$ and Euclidean ${\bar{R}}_{Eucl}$ averages, and how the calculation of the mean rotation vector $\bar{\varphi }$ leads to different results for values beyond the third decimal place. Based on these considerations, the mean rotation matrix was derived from the mean rotation vector $\bar{\varphi }$: the latter is calculated as the arithmetic mean of the rotation vectors $\varphi_{n}$.

As a second hypothesis, we tried to obtain the rigid transformation matrix Ta*o as above, but including in the calculation only one subset of the Ta,no from the initial dataset. This selection was made to eliminate outliers from the calculation of mean vectors $\bar{\varphi }$ and $\bar{t}$, as these values lead to a polarization of the results obtained from the application of the arithmetic mean to the vectors $\varphi_{n}$ and $t_{n}$. In particular, an algorithm was implemented in Matlab^®^ for the automatic determination of the set of matrices Ta,so with s≤n, to be included in the calculation of Ta*o. It operates in the following way:

1. for each Ta,io with *i* ∈ [1, n], if the RMSE_i_ (Root Mean Square Error calculated by applying the ICP algorithm in the fine registration stage) is less than RMSE*, than Ta,io ∈ Ta,ro, with r≤n, otherwise it is discarded;
2. for each Ta,ro, the translation vectors $t_{r}$ and the rotation vectors $\varphi_{r}$ are determined;
3. for each component *x_j_* of the vectors $t_{r}$ and $\varphi_{r}$ with *j* ∈ [1,3], it determines the interquartile range IQR(*x_j_*) (*i.e.*, the difference between the third *q_0,75_* and first quartile *q_0,25_* in the ordered series of data);
4. if (*x_j_* − *q_0,75_*) or (*q_0,25_* − *x_j_*) > 3 IQR(x_j_), then $t_{r}$*(x_j_)* (or $\varphi_{r}$*(x_j_)*) is discarded, otherwise $t_{r}$*(x_j_)* (or $\varphi_{r}$*(x_j_)*) ∈ $t_{s}$ (or $\varphi_{s}$);
5. calculates Ta*o from $t_{s}$ and $\varphi_{s}$ vectors as in the first algorithm.

The third proposed solution is based on an algorithm implemented in Matlab^®^, which automatically determines the final transformation matrix Ta*o, by selecting it from the Ta,no matrices included in the initial dataset. It operates in the following way:

1. for each Ta,no, it applies this transformation to the $P_{a,n}$ 3D acoustic point clouds, to align them with the corresponding 3D optical point clouds $P_{o,n}$;
2. for each pair $P_{o,n}P_{a,n}$, the mean distance $d_{oa,n}$ between the points of the 3D optical cloud and the corresponding of the acoustic 3D point cloud is calculated;
3. calculates $\bar{d_{n}}$ as the mean of $d_{oa,n}$;
4. selects the $\bar{d_{n,min}}$ as the minimum value of $\bar{d_{n}}$;
5. assumes as Ta*o the transformation Ta,no corresponding to $\bar{d_{n,min}}$.

## 3. Experimental Setup

### 3.1. System Configuration

**Figure 3:**
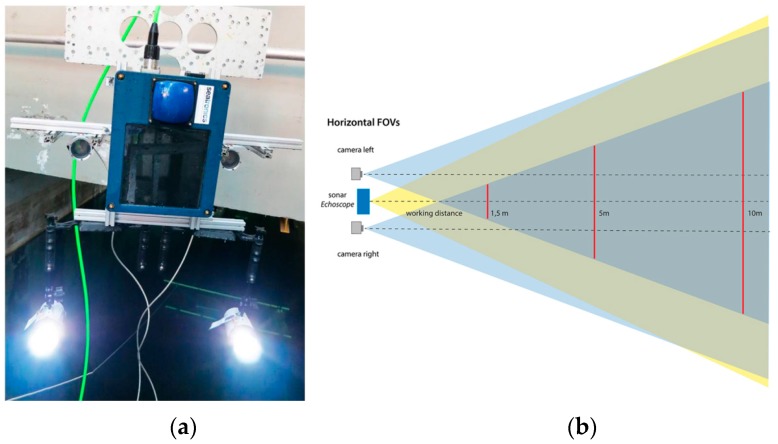
Layout of the opto-acoustic 3D camera (**a**). A scheme showing the overlap of the FOVs of Echoscope sonar and optical cameras (**b**).

The proposed system is composed of a stereo optical camera and an acoustic camera that will be attached to the ROV rigid frame. We used a Coda Echoscope camera for acoustic sensing. Through the ensonification of the whole viewing volume with a single ping, it uses the phased array technology to process approximately 16,000 beams simultaneously and generate a real-time 3D acoustic image of the entire observed scene. Concerning the optical component of the system, an optical stereo camera, consisting of two ultra-compact digital cameras housed in custom-made waterproof cases, has been developed. The system layout was defined through the use of a CAD model to ensure the maximum overlap of both FOVs at the minimum working distance (about 1 m, minimum working range of the Echoscope sonar) (Figure 3).

#### 3.1.1. Stereo Optical Camera

The stereo optical camera is the result of a research activity conducted at the Department of Mechanical, Energetic and Management Engineering (DIMEG) of the University of Calabria in the field of the underwater stereo photogrammetry, both for passive and active applications [3,30].

The stereo rig is composed of two ultra-compact digital cameras Point Grey Flea 2, with a Charge Coupled Device (CCD) sensor format of 1/3″, a resolution of 0.8 MP (pixel size of 4.65 μm) and a frame rate of 30 fps. The devices are also equipped with a pair of 8.5 mm Pentax C30811TH optical packages. 

Since the two cameras were not specifically made for the underwater environment, we have designed and constructed two waterproof housings. The body is made of aluminum to ensure efficient heat dissipation, while the flat port of the camera housing is made of polycarbonate. This solution leads to a reduction of the FOV caused by the refraction of the air-water interface, but its construction is easier. The camera is fixed within the case through an appropriate support that also works as a heat sink. 

The system is able to generate a 3D point cloud (about 200,000 points) at a frame rate of 7 fps. The Libelas library is used for the implementation of the stereo matching algorithm and the generation of the disparity map. We have verified that, in real-life conditions (*i.e.*, at sea), the Efficient LArge-scale Stereo (ELAS) algorithm [31] allows for obtaining a more robust and accurate 3D point cloud if compared to that obtained with the library OpenCV [32], which is used, instead, for the rectification of the stereo pair.

The main goal of the system is to improve the perception of the underwater scene by providing an output that enabling direct identification of individual objects (rocks, pipelines, walls, archeological artifacts). Their size ranges from centimeters to meters. To identify them with sufficient detail, almost 15 pixels per object are needed [33]. Therefore, a ground sample distance (GSD) less than 10 mm should be assured in the working volume and, consequently, an accuracy better than 10 mm has to be guaranteed. Taking into account the refractive effect of the medium on the camera parameters, the maximum operating distance should range from 8 m to 10 m (depending upon the quality of the acquired images). In this operating range, following previous works [34], we expect an operational accuracy from 0.2% to 0.7% for length measurements in real conditions.

#### 3.1.2. Acoustic Camera

Echoscope is a 3D acoustic camera that provides real-time, high angular resolution images of the acoustic environment. It consists of two distinct subsystems: one containing the acoustic units for transmitting and receiving signals (TX/RX unit), and another for processing the signals to be used in the beamforming process. The head of the acoustic camera has two physically distinct sections, TX array and RX array, both made with conventional piezoelectric sensors. 

The acoustic camera ensonifies the volume of observation through a single acoustic pulse and receives the energy reflected from an object that intercepts the propagation through a receiving array of hydrophonic sensors. The TX array for the generation of transmission signals is a wide-beam projector aimed to the ensonification of the environment. The transmission pulses are emitted with a frequency of 610 kHz (or 375 KHz) and a duration of 20 ms: these are generated with a repetition period between subsequent pulses equal to 100 ms, to ensure an adequate number of frames per second.

The receiving section, placed at the bottom of the acoustic camera, is a square planar array made of 48 × 48 analog sensors/channels. It is characterized by an acquisition range between 5 and 10 ms, with a sampling frequency of 10 MHz. Each one of the receiving channels will acquire 1024 samples, for a total of 2304 × 1024 samples. A beamforming process will be conducted on these samples for the generation of high angular resolution acoustic images. 

The working range of the Echoscope is from 1 m to 100 m, with a range resolution of 30 mm and a beam spacing of 0.19°. As stated by the manufacturer, the system meets, in real-time, the IHO S-44 Special Order Quality Surveys standard [35], with no post-processing of the point clouds. Therefore, we expect an accuracy of data less than 261 mm at a working distance of 10 m.

### 3.2. Laboratory Setup

**Figure 4:**
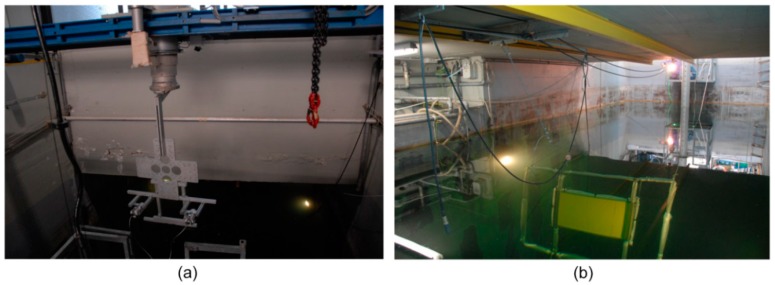
The telescopic pole for the handling of the opto-acoustic camera prototype (**a**). Conveyor belt system for the handling of the orientation fixture (**b**).

The alignment methodology has been tested at the electro-acoustic laboratory of the Whitehead Sistemi Subacquei S.p.A. (WASS) in Pozzuoli (Naples, Italy), equipped with a water tank for acoustic measurements (11 × 5 × 7 m) and the necessary tools for handling both the prototype of opto-acoustic camera (telescopic pole) and the target to be acquired (conveyor belt). To ensure a mechanical support for the system, a frame consisting of aluminum profiles has been designed and built. This frame houses the waterproof cases of the two optical cameras and the acoustic section of the Echoscope sonar, the latter connected through a rear plate. The entire structure was then fixed to a support bracket for a mechanical interfacing with a telescopic pole (Figure 4).

**Figure 5:**
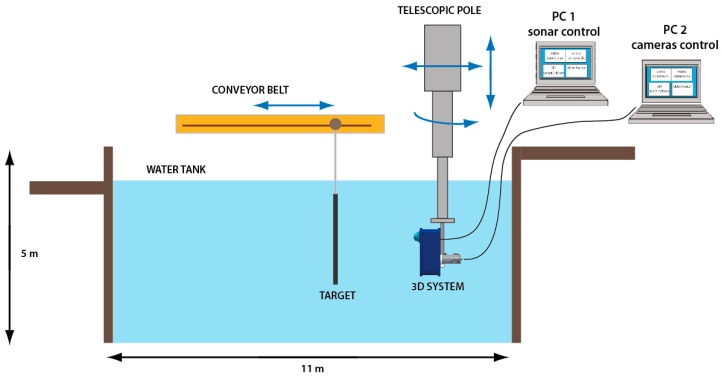
Experimental setup developed at the WASS electro-acoustic lab.

A schematic layout of the designed laboratory setup is depicted in Figure 5: it shows the connections between the 3D acquisition system (consisting of the acoustic section of the Echoscope sonar and the waterproof cases in which the two optical cameras are housed) and the PC, and the various systems for the handling of the target and the opto-acoustic camera itself.

The acoustic section is connected, via a proprietary bus, to a Power Supply Unit (PSU), while the workstation (which hosts the software for the management of the entire acoustic subsystem, *i.e.*, the Data Integration Unit or DIU that manages the flow of data coming from the acoustic section with the related I/O controls, and the software UIS Survey Explorer for the visualization of the acoustic 3D point cloud) is connected via a RS232 port (sending and receiving commands) and an Ethernet 10 Mbps interface (receiving data). In turn, the stereo optical subsystem is connected to a PC that allows for the management and the configuration of the acquisition parameters via a Firewire 800 interface, by means of the Flycapture software. The synchronization of the stereoscopic camera is carried out through the trigger function of this software.

The opto-acoustic system is fixed to a telescopic pole that allows for its immersion in the water tank and offers the possibility of handling it within the four degrees of freedom shown in the diagram, while the target to be acquired is fixed to a conveyor belt that allows for its longitudinal translation.

**Figure 6:**
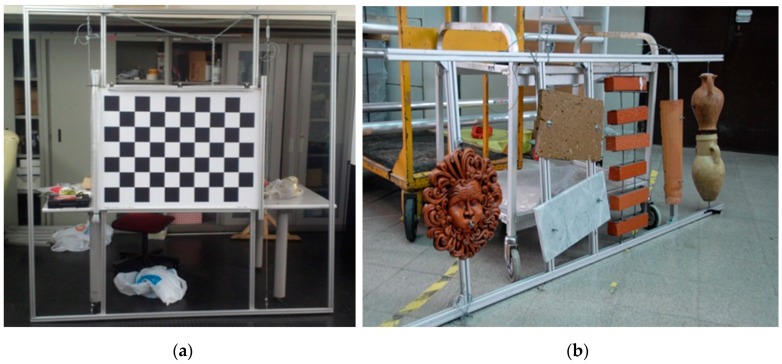
Orientation fixture (**a**). Target made of objects with different materials and shapes (**b**).

Figure 6 shows the targets used in various experimental phases. Their sizes were determined through the analysis of the FOV of the two subsystems in the operating range. In addition to the orientation fixture (an aluminum frame of 2 m × 2.5 m with a central checkerboard of 9 × 7 squares, each one with a size of 100 × 100 mm), we built an additional panel with objects of different shapes and materials. In particular, we can see two ceramic vases, marble and tufa tiles, bricks, roofing tiles, and a mask made of terracotta.

## 4. Experimentation

In this Section we will describe the operations carried out at the different stages of the planned series of tests, to evaluate the performance of the designed opto-acoustic camera prototype. The purpose of the tests is twofold: on the one hand, we want to evaluate which one of the three statistical processing methods ends up to be more effective; on the other hand, we want to get a validation of the alignment method. This has been obtained by estimating the mean distance μ (and the respective standard deviation σ) among the aligned optical and acoustic 3D point clouds of the target panel.

During the selection of the most efficient methodology to be adopted for the calculation of the matrix Ta*o, as discussed in Section 2.3 by comparing the implementation hypotheses, we would assess the complexity of the adopted solution and the quality of the obtained results, considering that there is a limit to the accuracy of the registration of the acoustic and optical 3D point clouds. This limit is due to the error that occurs in the reconstruction of the 3D point cloud by the stereo optical system and the accuracy of the acoustic camera in the reconstruction of the rig. In fact, any result below this threshold would be completely random and linked to the specific dataset used to determine the matrix Ta*o.

### 4.1. Image Acquisition

Prior to the actual acquisition stage, we carried out some operations to configure the optical and acoustic sensors of the system. In particular, concerning the stereo optical subsystem, we adjusted the focus settings for the cameras in air and performed some acquisition tests underwater, to verify the correct superposition of the FOVs, as provided for in the design of the support frame.

**Figure 7:**
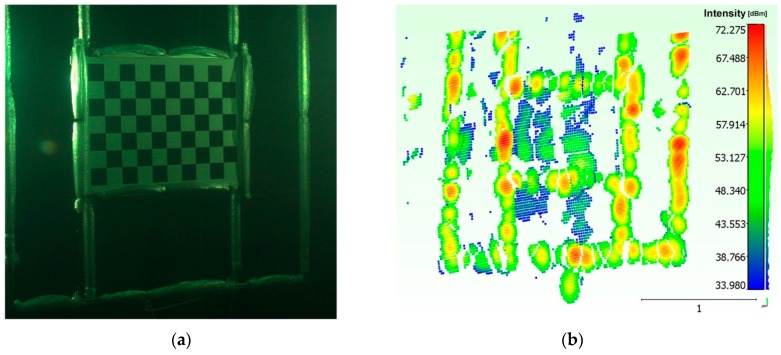
Acquisition of a pose of the orientation rig: (**a**) optical; (**b**) acoustic.

The Echoscope sonar has been adjusted to excite the transmission section with the highest working frequency, corresponding to 610 KHz. In this stage we found that the optical axes had to be kept slightly convergent rather than parallel, to correct the overlap of the FOVs of the two cameras in the range of operation. As for the alignment methodology, we acquired a sequence of 20 different poses of the orientation fixture, by positioning it at a distance varying from about 1.5 m to 10 m and in different orientations. The panel was rolled, tilted and twisted to reduce the correlation of the calibration parameters of the optical stereo camera, while the opto-acoustic camera was clamped to a telescopic pole varying its transverse position and orientation (Figure 7).

**Figure 8:**
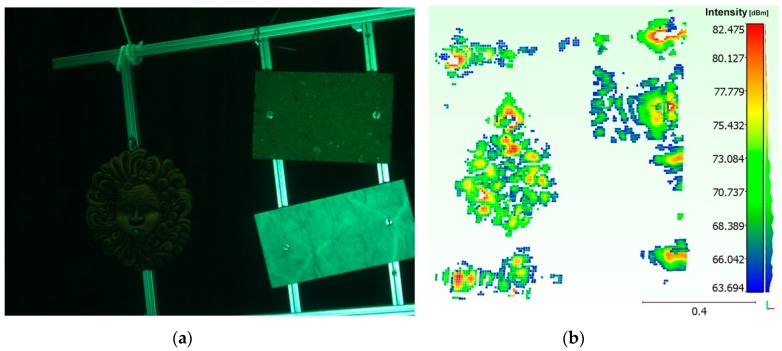
Target acquisition with objects: (**a**) optical; (**b**) acoustic.

Three different poses of the panel containing objects of various shapes and materials were acquired (Figure 8). The panel was positioned on the side edge of the water tank (at a distance of approx. 2.5 m from the opto-acoustic camera) and the camera was handled by means of the telescopic pole.

The output of the acoustic camera, which—in addition to the image of the acquired 3D point cloud—shows the scalar field representing the intensity of the echoes received for each acquired point, has immediately shown the strong limits of Echoscope in close range applications. In fact, contrary to what is stated by the manufacturer, the sonar was not able to process correctly the scattering signal returned by the objects placed at distances less than 2.5 m. This could be due to two causes: either an excessive duration of the transmitted pulse (a pulse with a duration of 2 ms generates a blind range equal to 2 m) or, more likely, the type of beamforming applied, which does not allow for an appropriate phase correction for focusing at close range.

### 4.2. Data Processing

**Figure 9:**
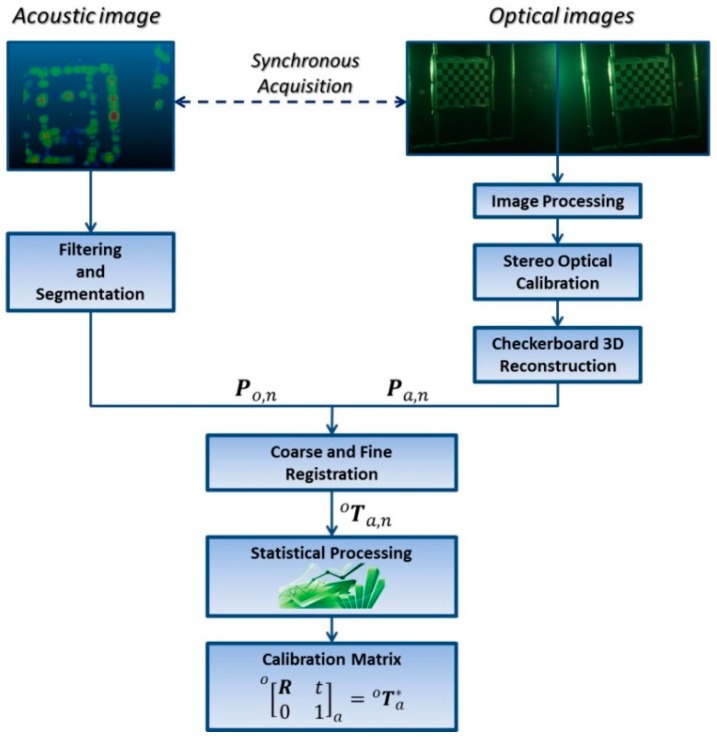
Processing pipeline of acoustic and optical data.

The processing pipeline of the data acquired through the optical and acoustic sensors is shown in Figure 9. Starting from the synchronous acquisition of the *n* poses of the orientation rig during the early stage of the process, the system outputs *n* pairs of 3D point clouds, where the *n-*th pair is formed by the optical $P_{o,n}$ and the acoustic $P_{a,n}$ 3D point clouds, to calculate, by means of coarse and fine registration technique, *n* estimates Ta,no of the rigid transformation matrix.

At the end of the process, as described in Section 2.3, the final transformation matrix Ta*o is obtained by statistically processing the dataset composed of *n* transformation matrices Ta,no obtained downstream of the previous registration stage.

#### 4.2.1. Acoustic Image Processing

The 3D data provided by the acoustic camera can be corrupted either by false reflections caused by the secondary lobes of the receiving array or by the noise present in the acquisition phase of the backscattering signals. Although the Echoscope directly performs a low-level preprocessing, it is still necessary to conduct some appropriate operations on the acoustic 3D point cloud, to clean up the images from noise or potential outliers. So it is evident that the operations of filtering (noise reduction and the elimination of possible outliers) and segmentation (differentiation of objects and background in the observed scene) are to be considered as preliminary and mandatory steps for the execution of all integration algorithms to be applied to this specific type of data [36,37].

The solution adopted in this first implementation of the alignment method is based on a thresholding method, performed through the open source software CloudCompare [38].

Given the range and intensity information provided by the acoustic camera, we used two threshold tests to discriminate between actual backscattered echoes and clutters. The method operates in the following way:

1. Test 1: assuming a Gaussian distribution for echo amplitude noise, 3D points with intensity lower than a threshold *thr_1_* are discarded, while the others are considered as belonging to the target;
2. Test 2: given the histogram of the range values and knowing the geometry of the rig, 3D points outside an interval defined by the thresholds *thr_2_* and *thr_3_* are discarded, while the others are considered as belonging to the target.

**Figure 10:**
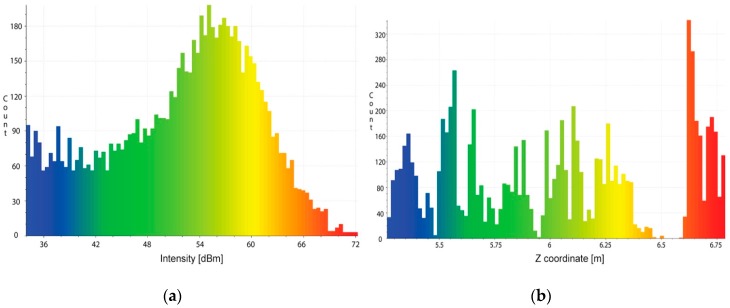
Distribution of the echo intensity (**a**), histogram of the range values (**b**).

Threshold values are manually defined, through a vision inspection of the echo and range distribution, respectively (Figure 10), as the implementation of an automated procedure would require further, more focused research. However, different automatic thresholding and filtering methods are described in the literature. A comprehensive review is reported in [36].

**Figure 11:**
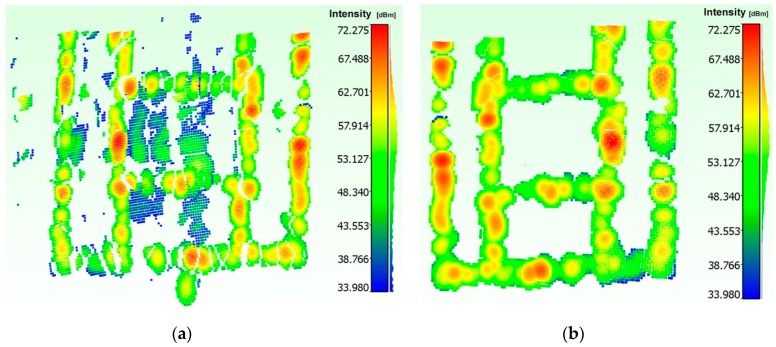
Acoustic 3D point cloud: before (**a**) and after filtering and segmentation stage (**b**).

Applying the above thresholding method, we have obtained the results shown in Figure 11.

#### 4.2.2. Stereo Optical Image Processing

To obtain the 3D point clouds of the orientation rig for each pose assumed during the acquisition stage, the stereo optical images are processed through the algorithms reported in Section 3.1.2.

Before 3D reconstruction, the underwater images have been pre-processed to reduce blur due to scattering effects and correct color casts (remove greenish-blue components). This can be done through two different approaches: digital restoration techniques or methods of image enhancement [39,40]. In the proposed alignment method, the adopted solution provides for an image enhancement methodology based on the technique presented in [41].

**Figure 12:**
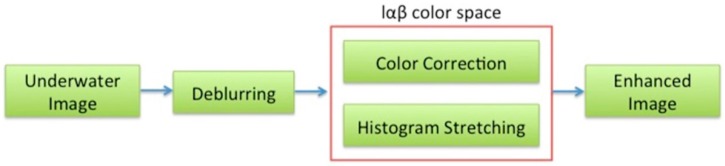
Flow-chart of the image enhancement steps.

As a first step, sharp filtering has been performed to remove the fog in the images, due to the scattering effects, which decreased image contrast and increased the blur. Secondly, the images are color corrected through a white balancing in the lαβ color space. It allows for removing the color casts in underwater images that typically suffer from color alterations, by balancing the chromatic components (α and β), while the luminance component (l) is used to improve image contrast by cut-off and histogram stretching (Figure 12). The method is particularly suitable to process our datasets, as it has been proposed for applications like close-range acquisition in nadir direction.

**Figure 13:**
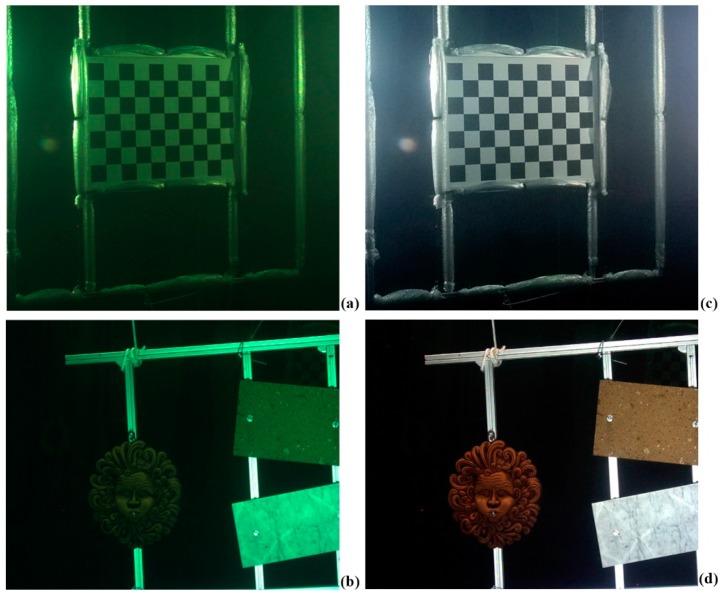
Optical images of the orientation rig (**a**) and target objects; (**b**) after the application of the color enhancement algorithm (**c**,**d**).

Figure 13 shows the results of the algorithm applied to the optical images shown in Figure 7 and Figure 8, respectively.

**Figure 14:**
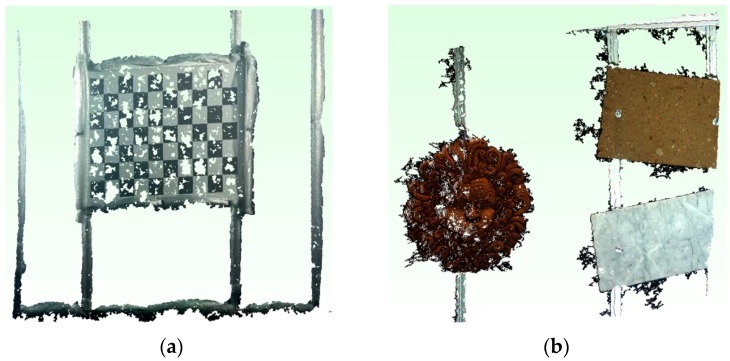
Optical 3D reconstruction of the orientation rig (**a**) and the target with objects (**b**).

Subsequently, the acquired optical images have been processed to generate the 3D point clouds of the orientation rig and the panel with objects in various poses. The application of the algorithms has allowed for the reconstruction of 11 over 20 poses of the orientation frame, since in 9 poses the stereo matching algorithm was not able to reconstruct the target. A stage of manual cleaning of the raw 3D point clouds performed with CloudCompare, downstream of the reconstruction process, was necessary to eliminate several outlier points. Automatic methods can be used for this stage. For example, the algorithms present in the Point Cloud Library (PCL) [42]. Figure 14 shows two examples of optical 3D point clouds of the orientation frame and the target, respectively.

#### 4.2.3. Optical and Acoustic Registration

**Figure 15:**
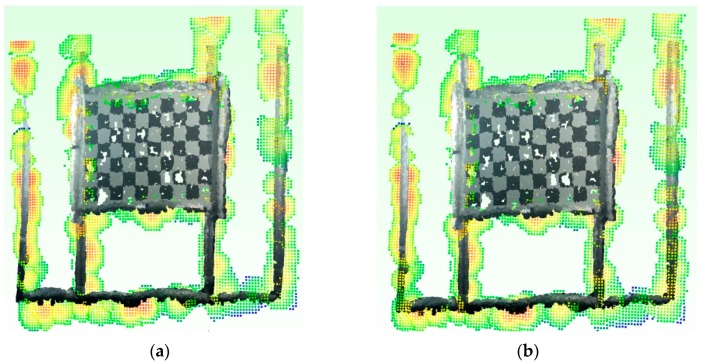
Optical and acoustic 3D point clouds after coarse (**a**) and fine; (**b**) registration stages.

As described in Section 2.2, the relative orientation between the optical and acoustic system is obtained downstream of a statistical processing of Ta,no estimates coming from the registration of the *n* pairs $P_{o,n}P_{a,n}$ of optical and acoustic 3D point clouds. This registration stage was performed for each of the pairs $P_{o,n}P_{a,n}$ with *n* ∈ [1,11] derived from the previous step of the implemented alignment method. Figure 15 shows the results of the algorithms on one of the 11 acquired pairs.

#### 4.2.4. Statistical Processing of the Transformation Matrices

The different methods of statistical processing described in Section 2.3 were applied on the dataset composed of Ta,no with *n* ∈ [1,11] estimates of the unknown rigid transformation matrix, obtaining three approximations of the Ta*o matrix, *i.e.*, Ta,1*o, Ta,2*o, Ta,3*o respectively.

**Figure 16:**
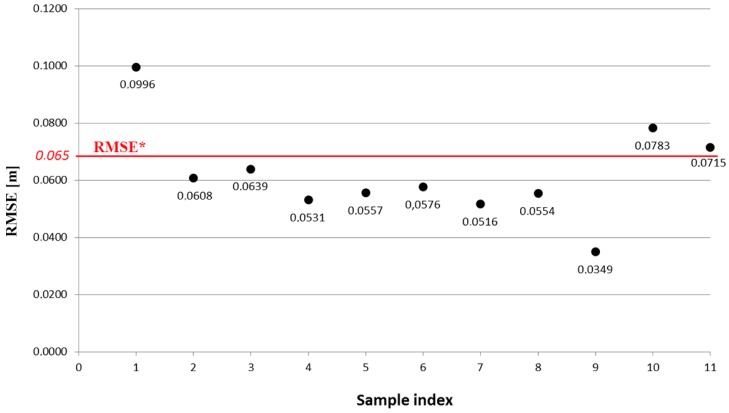
Graph of RMSE values obtained downstream of the registration process (Section 4.2.3) on $P_{0,n}P_{a,n}$ pairs of 3D point clouds.

Concerning the second methodology, the choice of the RMSE* value was carried out by analyzing the graph in Figure 16, that shows the RMSE values associated with each of the $P_{o,n}P_{a,n}$ pairs of registered 3D point clouds. These were ranked in ascending order along the *x*-axis, as a function of the acquisition distance by the opto-acoustic imaging system. As can be seen from the graph, the pairs $P_{o,n}P_{a,n}$ with *n* = 1, 10, 11 present a higher RMSE value than the remaining ones, so the RMSE* was set to 65 mm, to eliminate the related matrices Ta,no from the average calculation.

By applying this solution, five of the 11 estimates Ta,no of the rigid transformation matrix have been discarded. It is interesting to note the existing relation among the RMSE values of the ICP algorithm and the different poses of the rig. The sample with index *i* = 1 in the graph (Figure 16) is associated with the only pair of 3D point clouds $P_{o,1}P_{a,1}$ that we were able to obtain from the first nine poses of the orientation frame over a total of 20 poses. These have been acquired at a distance of between 1.5 m and 4.5 m from the opto-acoustic camera. The samples with index *i* ∈ [2,9] refer instead to the eight poses acquired at a distance of between 4.5 m and 7 m. Finally, the samples with index *i* = 10, 11 refer to the pairs of 3D point clouds $P_{o,10}P_{a,10}$ and $P_{o,11}P_{a,11}$, obtained from two of the three final poses acquired at a distance of between 7 m and 10 m. The pairs with high RMSE values are associated with the poses acquired at a distance of less than 4.5 m and more than 7 m. Thus, we can deduce that the optimal condition for the opto-acoustic camera, with respect to the specific target, is within distances of between about 4.5 m and 7 m. As one can expect, at a distance of less than 4.5 m, the acoustic component of the system presents the main limitations in reconstructing the acquired target, while for distances greater than 7 m, the stereo optical camera suffers from poor performance, as the stereo algorithm fails to process the acquired images.

## 5. Results

**Figure 17:**
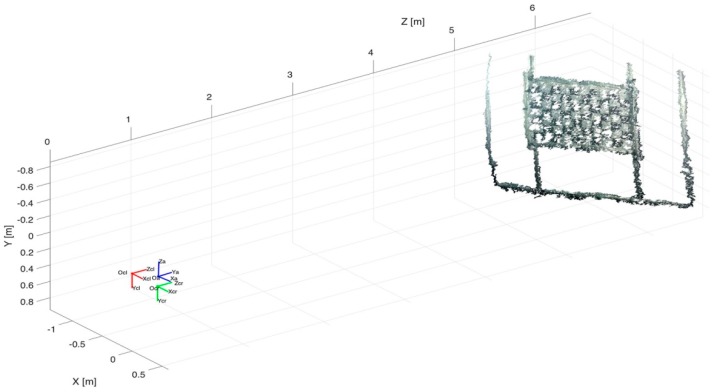
Relative pose of the optical cameras and Echoscope coordinate systems for one pose of the orientation frame.

From the three statistical processes we have estimated the rigid transformation matrix that is needed to perform the relative orientation of the opto-acoustic system. Figure 17 shows the relative positions of the coordinate systems related to each device, for one pose of the orientation frame.

Table 1 shows the Euler angles computed by the rotation matrix and the norm of the translation vector for each of the three methods. We should notice that there is a relevant difference among the values calculated by the three methods and, in particular, for the magnitude of the translation.

**Figure 18:**
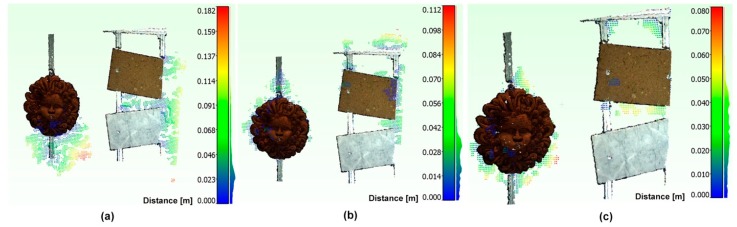
Alignment of opto-acoustic 3D point clouds of the panel with objects, obtained through the application of matrices Ta,1*o (**a**), Ta,2*o (**b**), Ta,3*o (**c**).

We have analyzed the obtained results, in terms of mean registration error, by applying the three transformation matrices on the pair of optical and acoustic 3D point clouds belonging to the panel with objects (Figure 18). Table 2 reports the mean registration error μ and the standard deviation σ.

The data confirm that the third statistical method provides the best results. As can be noted in Figure 18c, the values with the greater distance are due to the noise of the acoustic 3D point cloud, especially in proximity of the terracotta mask and the aluminum frame of the panel itself.

To define the accuracy of each system, we consider as ground truth the known-size of the orientation frame. In particular, for the optical system we obtained a value of 6.45 mm as RMSE computed by measuring the diagonals of the checkerboard (of which the dimensions were known) for all poses on the optical point clouds. This is equivalent to a length accuracy of 0.4%. The same procedure was followed for the acoustic system, knowing the external dimensions of the aluminum frame (2 × 2.5 m), and measuring them on the 3D acoustic point clouds for each pose. In the latter case we obtain a RMSE of 15 mm, equivalent to a length accuracy of 0.75%. The obtained results are consistent with the expected ones.

To finalize the validation, we have computed the accuracy of the integrated system by measuring several known lengths on the opto-acoustic 3D point clouds. In this case we have obtained a RMSE of 17.8 mm.

**Figure 19:**
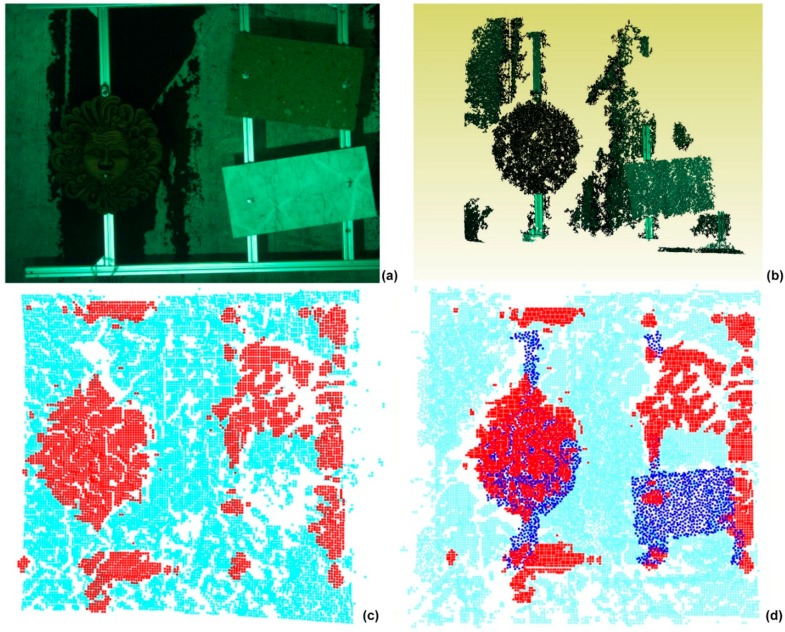
A pose of the target with objects (**a**), optical 3D point cloud; (**b**), acoustic point cloud; (**c**), opto-acoustic reconstruction of the scene (**d**).

The effectiveness and the potentiality of the system can be further evaluated by analyzing the image in Figure 19. This was obtained by integrating off-line the low resolution acoustic 3D point cloud with the high resolution optical cloud of the acquired scene (Figure 19a). Due to the different materials, the marble tile was present only in the optical acquisition (Figure 19b) while the tufa tile was featured only in the acoustical one (Figure 19c). The integration of the acoustic point cloud (red points) with the optical point cloud (blue points) allows for compensating the errors in the full field-acquisition of the acoustic camera and better discriminate the objects from the background (sky blue points).

## 6. Summary, Discussion and Outlook

This paper presents a first step towards the realization of an opto-acoustic system for the 3D imaging of the underwater scenes. The system is composed of a stereo optical camera and a 3D acoustic camera and is designed to be installed on a ROV, to improve its control capabilities by the operator during the navigation in turbid water conditions or in close range manipulation operations. The presented opto-acoustic camera is the first underwater systems employing two 3D imaging sensors to obtain a 3D representation of the underwater scene.

In the current work, a multi-sensor registration method has been proposed for the automatic integration of 3D data acquired from the two systems, specifically for this application. We have solved the challenging problem to determine automatically a correspondence between the two different data types, by means of an orientation frame that is able to highlight some geometric features detected from both sensors. Due to the low resolution and the high dispersion of acoustic data, a statistical processing has been performed on several estimations of the geometric transformation that allows for aligning the two datasets. The statistical approach increases the robustness of the estimation by reducing the effect of the noise component. Moreover, we have described in detail the processing stages needed to prepare the data to be integrated. The results demonstrate that the presented methodology is able to return a 3D image made of integrated data. This calls for further investigations in real conditions.

The comparison of technical details and performance capability with other systems based on this technique is not an easy task. To the best of the authors’ knowledge, few works in the literature present an evaluation of the registration accuracy of the 3D opto-acoustic data. In [20], an average error of 24.2 mm is obtained when computing the distance of all the reprojected multibeam points to their respective calibration planes while, in [12], the authors report an average registration error between the 3D acoustic data and the CAD model of a pipe of approximately 70 mm. Finally, in [14] the estimated 3D points are within 3.5% of their distances to the optical cameras, utilizing the reconstruction of a plane as ground truth. A maximum error of 63 mm is obtained. These results show how the integration accuracy of our alignment method, as reported in Section 5, has the same magnitude.

The main challenges for the future developments of such a system will be: (a) the search for a 3D acoustic camera that can provide a better performance in the close range to obtain better data on the operating volume of the ROV; (b) the implementation of on-line data integration and visualization techniques, to make the ROV operator able to interpret in the best way the data generated by the integration of optical and acoustic sensors; (c) conducting experimental tests in real conditions.

## Figures and Tables

**Table 1:** Euler angles and norm of the translation vector for the three statistical methods.

	I Method	II Method	III Method
α (°)	89.763	87.498	88.394
β (°)	−6.771	−6.154	−5.549
γ (°)	2.869	1.268	0.276
Norm(t) (mm)	0.4920	0.3188	0.2718

**Table 2:** Mean error of the optical and acoustic registration of the target with objects.

	I Method	II Method	III Method
μ (mm)	46.2	23.4	18.2
σ (mm)	36.5	14.3	13.7

## References

[1] Johnson-Roberson M., Pizarro O., Williams S.B., Mahon I. (2010). Generation and visualization of large-scale three-dimensional reconstructions from underwater robotic surveys. J. Field Robot..

[2] Drap P., Merad D., Boï J.M., Mahiddine A., Peloso D., Chemisky B., Seguin E., Alcala F., Bianchimani O. (2014). Underwater multimodal survey: Merging optical and acoustic data. Underwater Seascapes.

[3] Bruno F., Bianco G., Muzzupappa M., Barone S., Razionale A.V. (2011). Experimentation of structured light and stereo vision for underwater 3D reconstruction. ISPRS J. Photogramm. Remote Sens..

[4] Singh H., Salgian G., Eustice R., Mandelbaum R. Sensor fusion of structure-from-motion, bathymetric 3D, and beacon-based navigation modalities. Proceedings of the ICRA’02 International Conference on Robotics and Automation.

[5] Snavely N., Seitz S.M., Szeliski R. (2008). Modeling the world from internet photo collections. Int. J. Comput. Vis..

[6] Dissanayake M.G., Newman P., Clark S., Durrant-Whyte H.F., Csorba M. (2001). A solution to the simultaneous localization and map building (SLAM) problem. IEEE Trans. Robot. Autom..

[7] Williams S., Mahon I. Simultaneous localisation and mapping on the great barrier reef. Proceedings of the ICRA’04 International Conference on Robotics and Automation.

[8] Kunz C., Singh H. (2013). Map building fusing acoustic and visual information using autonomous underwater vehicles. J. Field Robot..

[9] Grisetti G., Kummerle R., Stachniss C., Burgard W. (2010). A tutorial on graph-based SLAM. IEEE Intell. Transp. Syst. Mag..

[10] Fusiello A., Giannitrapani R., Isaia V., Murino V. Virtual environment modeling by integrated optical and acoustic sensing. Proceedings of the Second International Conference on 3-D Digital Imaging and Modeling.

[11] Fusiello A., Murino V. (2000). Calibration of an optical-acoustic sensor. Mach. Graph. Vis..

[12] Fusiello A., Murino V. (2004). Augmented scene modeling and visualization by optical and acoustic sensor integration. IEEE Trans. Vis. Comput. Graph..

[13] Hansen R.K., Andersen P.A. (1996). A 3D underwater acoustic camera—properties and applications. Acoustical Imaging.

[14] Negahdaripour S., Sekkati H., Pirsiavash H. (2009). Opti-acoustic stereo imaging: On system calibration and 3-D target reconstruction. IEEE Trans. Image Process..

[15] Negahdaripour S., Pirsiavash H., Sekkati H. Integration of Motion Cues in Optical and Sonar Videos for 3-D Positioning. Proceedings of the CVPR’07 Conference on Computer Vision and Pattern Recognition.

[16] Sekkati H., Negahdaripour S. Direct and indirect 3-D reconstruction from opti-acoustic stereo imaging. Proceedings of the Third International Symposium 3D Data Processing, Visualization, and Transmission.

[17] Negahdaripour S. Calibration of DIDSON forward-scan acoustic video camera. Proceedings of the MTS/IEEE OCEANS 2005.

[18] Negahdaripour S. (2007). Epipolar geometry of opti-acoustic stereo imaging. IEEE Trans. Pattern Anal. Mach. Intell..

[19] Nicosevici T., Garcia R. Online robust 3D mapping using structure from motion cues. Proceedings of the MTS/IEEE OCEANS 2008.

[20] Hurtós N., Cufì X., Salvi J. Calibration of optical camera coupled to acoustic multibeam for underwater 3D scene reconstruction. Proceedings of the OCEANS 2010 IEEE-Sydney.

[21] Zhang Q., Pless R. Extrinsic calibration of a camera and laser range finder (improves camera calibration). Proceedings of the IEEE/RSJ International Conference on Intelligent Robots and Systems (IROS 2004).

[22] Besl P.J., McKay N.D. (1992). Method for registration of 3-D shapes. IEEE Trans. Pattern Anal. Mach. Intell..

[23] Lowe D.G. (1991). Fitting parameterized three-dimensional models to images. IEEE Trans. Pattern Anal. Mach. Intell..

[24] Castellani U., Fusiello A., Murino V., Papaleo L., Puppo E., Pittore M. (2005). A complete system for on-line 3D modelling from acoustic images. Signal Proc. Image Commun..

[25] Huang Y., Qian X., Chen S. (2009). Multi-sensor calibration through iterative registration and fusion. Comput. Aided Des..

[26] Heikkila J. (2000). Geometric Camera Calibration Using Circular Control Points. IEEE Trans. Pattern Anal. Mach. Intell..

[27] Zhang Z. (2000). A flexible new technique for camera calibration. IEEE Trans. PAMI.

[28] Shortis M. (2015). Calibration Techniques for Accurate Measurements by Underwater Camera Systems. Sensors.

[29] Sharf I., Wolf A., Rubin M.B. (2010). Arithmetic and geometric solutions for average rigid-body rotation. Mech. Mach. Theory.

[30] Bianco G., Gallo A., Bruno F., Muzzupappa M. (2013). A Comparative Analysis between Active and Passive Techniques for Underwater 3D Reconstruction of Close-Range Objects. Sensors.

[31] Geiger A., Roser M., Urtasun R. (2011). Efficient Large-Scale Stereo Matching.

[32] OpenCV. http://opencv.org/.

[33] Comer R., Kinn G., Light D., Mondello C. (1998). Talking Digital. Photogramm. Eng. Remote Sens..

[34] Barnes H. (2003). Oceanography and Marine Biology.

[35] Mills G.B. (2015). International hydrographic survey standards. Int. Hydrogr. Rev..

[36] Murino V., Trucco A. (2000). Three-dimensional image generation and processing in underwater acoustic vision. IEEE Proc..

[37] Murino V. (2001). Reconstruction and segmentation of underwater acoustic images combining confidence information in MRF models. Pattern Recognit..

[38] CloudCompare. http://www.danielgm.net/cc/.

[39] Schettini R., Corchs S. (2010). Underwater image processing: State of the art of restoration and image enhancement methods. EURASIP J. Adv. Signal Process..

[40] Mahiddine A., Seinturier J., Boi D.P.J., Drap P., Merad D., Long L. Underwater image preprocessing for automated photogrammetry in high turbidity water: An application on the Arles-Rhone XIII roman wreck in the Rhodano river, France. Proceedings of the 18th International Conference Virtual Systems and Multimedia (VSMM).

[41] Bianco G., Muzzupappa M., Bruno F., Garcia R., Neumann L. (2015). A New Color Correction Method for Underwater Imaging. ISPRS Int. Arch. Photogramm. Remote Sens. Spat. Inf. Sci..

[42] PointCloudLibrary (PCL). http://www.pointclouds.org/.

